# Spatiotemporal distribution of COVID-19 during the first 7 months of the epidemic in Vietnam

**DOI:** 10.1186/s12879-021-06822-0

**Published:** 2021-10-30

**Authors:** Toshie Manabe, Dung Phan, Yasuhiro Nohara, Dan Kambayashi, Thang Huu Nguyen, Thanh Van Do, Koichiro Kudo

**Affiliations:** 1 Nagoya City University Graduate School of Medicine, 1 Kawasumi, Mizuho-cho, Mizuho-ku, Nagoya, Aichi 467-8601 Japan; 2 Nagoya City University West Medical Center, Aichi, Japan; 3 Faculty of Pharmacy and Pharmaceutical Sciences, Monash University, Melbourne, VIC Australia; 4 Utsunomiya University Center for Regional Design, Tochigi, Japan; 5 Showa Pharmaceutical University Center for Education and Research on Clinical Pharmacy, Tokyo, Japan; 6 School for Preventive Medicine and Public Health, Hanoi Medical University, Hanoi, Vietnam; 7 Center for Tropical Diseases, Bach Mai Hospital, Hanoi, Vietnam; 8 Yurin Hospital, Tokyo, Japan; 9 Waseda University, Tokyo, Japan

**Keywords:** COVID-19, Emerging infectious disease, Spatiotemporal analysis, Disease clustering, Nosocomial infection

## Abstract

**Background:**

Understanding the spatiotemporal distribution of emerging infectious diseases is crucial for implementation of control measures. In the first 7 months from the occurrence of COVID-19 pandemic, Vietnam has documented comparatively few cases of COVID-19. Understanding the spatiotemporal distribution of these cases may contribute to development of global countermeasures.

**Methods:**

We assessed the spatiotemporal distribution of COVID-19 from 23 January to 31 July 2020 in Vietnam. Data were collected from reports of the World Health Organization, the Vietnam Ministry of Health, and related websites. Temporal distribution was assessed via the transmission classification (local or quarantined cases). Geographical distribution was assessed via the number of cases in each province along with their timelines. The most likely disease clusters with elevated incidence were assessed via calculation of the relative risk (RR).

**Results:**

Among 544 observed cases of COVID-19, the median age was 35 years, 54.8% were men, and 50.9% were diagnosed during quarantine. During the observation period, there were four phases: Phase 1, COVID-19 cases occurred sporadically in January and February 2020; Phase 2, an epidemic wave occurred from the 1st week of March to the middle of April (Wave 1); Phase 3, only quarantining cases were involved; and Phase 4, a second epidemic wave began on July 25th, 2020 (Wave 2). A spatial cluster in Phase 1 was detected in Vinh Phuc Province (RR, 38.052). In Phase 2, primary spatial clusters were identified in the areas of Hanoi and Ha Nam Province (RR, 6.357). In Phase 4, a spatial cluster was detected in Da Nang, a popular coastal tourist destination (RR, 70.401).

**Conclusions:**

Spatial disease clustering of COVID-19 in Vietnam was associated with large cities, tourist destinations, people’s mobility, and the occurrence of nosocomial infections. Past experiences with outbreaks of emerging infectious diseases led to quick implementation of governmental countermeasures against COVID-19 and a general acceptance of these measures by the population. The behaviors of the population and the government, as well as the country’s age distribution, may have contributed to the low incidence and small number of severe COVID-19 cases.

**Supplementary Information:**

The online version contains supplementary material available at 10.1186/s12879-021-06822-0.

## Background

In December 2019, a novel virus causing coronavirus disease 2019 (COVID-19) in humans emerged in Wuhan City, Hubei Province, China [1] and rapidly spread globally leading to a pandemic [2]. The virus was initially called the 2019 novel coronavirus (2019-nCoV) and was later renamed severe acute respiratory syndrome coronavirus-2 (SARS-CoV-2). As of the end of July 2020, more than 17 million infections had been reported in over 220 countries and regions globally [3]. However, numbers of COVID-19 cases and deaths differed enormously among countries and regions [3]. In Vietnam, the first two cases of COVID-19 were identified on 23 January 2020 in two Chinese nationals visiting Vietnam for the Lunar New Year holiday festival [4, 5]. Subsequently, local cases of COVID-19 occurred sporadically. As of 31 July 2020, 509 cases had been confirmed throughout the country with no deaths. By contrast, over 4.3 million and 2.5 million cases occurred in the USA and Brazil, respectively, with case fatality rates of approximately 3.5% in both countries [3]. The pandemic situation in Vietnam is significantly different from that in other countries, such as the USA, Brazil, Mexico, Russian, the UK, and Italy [3]. Although several factors may have contributed to this difference, the strong measures implemented by Vietnam to interrupt SARS-CoV-2 transmission at an early stage, including containment and closure of borders with entry bans [6], may provide part of the explanation. However, because Vietnam shares a long northern border with China as well as borders with Laos and Cambodia, the country may be at higher risk of transmission. As a resource-limited developing country, it is crucial for Vietnam to effectively track the COVID-19 pandemic.

The term “disease clustering” is used if the frequency of disease is higher than normally expected for a specific time or area adjusted by the population size. The situation of infection in the neighboring areas is also used for calculation. In such cases, the disease is considered to have clustered in that time or area [7]. It is also contributed to understand the spatial and temporal patterns of COVID-19 outbreaks. It helps the decision-making to determine the allocation of limited medical and human resources and the preparedness for the infection control measures, especially for developing countries. Detection of disease clustering contributes to identification of risk factors for exposure [8] as well as identification of the signals of a pandemic [9]. Several previous studies using spatiotemporal analysis have contributed to decision-making during outbreaks of emerging infectious diseases such as severe acute respiratory syndrome (SARS) [10], Middle East respiratory syndrome [11], avian influenza A (H5N1) [12] and (H7N9) viruses [13] infections and COVID-19 [14]. Thus, we hypothesized that data on the spatiotemporal distribution of COVID-19 in Vietnam could help in understanding the causes of the low numbers of COVID-19 cases observed in Vietnam and potentially help in the implementation of global control measures.

The aim of the present study was to evaluate the spatiotemporal distribution of COVID-19 in Vietnam during the first 7 months of the COVID-19 pandemic.

## Methods

We evaluated the spatiotemporal distribution of COVID-19 in Vietnam starting from 23 January 2020, the date that the first cases of COVID-19 were identified in Vietnam, until 31 July 2020. Daily data on laboratory-confirmed COVID-19 cases were collected from the situation reports of the World Health Organization (WHO) [15], the Vietnam Ministry of Health [16], and other websites.

The population of each province was obtained from a 2019 report by the General Statistics Office of Vietnam [17] and was used to calculate the standardized incidence ratio (SIR) for evaluating disease clustering [7, 18]. Geographical information, including the latitude and longitude of each province and its neighboring provinces, was also collected for disease mapping. The collected data for all cases were summed over 7 days (Monday to Sunday) to yield 1-week time periods (total 12 weeks). Weekly data were used to evaluate chronological differences in the number of reported cases and COVID-19 clustering. During the observation period, there were four phases of the COVID-19 epidemic in Vietnam. The first phase was the sporadic occurrence of COVID-19 in January and February 2020. The second phase was the first epidemic wave that occurred from the 1st week of March until the middle of April, mainly in Hanoi and Ho Chi Minh City (HCMC). The third phase involved only quarantined cases and no local cases. The fourth phase was from 25 July to the end of July and included the second epidemic wave.

### Statistical analyses

Disease clustering was assessed using a flexibly shaped space–time scan statistic (FleXScan) [7, 18, 19]. The FleXScan analysis was implemented using the restricted likelihood ratio proposed by Takahashi and Tango [18]. As the relative risk (RR) of the cluster becomes large via a Monte Carlo simulation, this method can detect clusters of any shape reasonably well [18, 19]. We selected a flexible spatial scan as our scanning method. A Poisson model was used for spatial analysis scanning and clustering; *p* values were calculated using the original log likelihood ratios. We estimated the SIR to detect spatial clusters. The SIR is the observed number of cases divided by the expected frequency of incidence within clusters. For the circulating spatial scan statistic, the maximum length of the geographical window was set to 15 of the nearest neighbors [18]. The number of replicates for the Monte Carlo procedure was set to 999.

Data analyses were conducted using FleXScan version 3.1.2 (National Institute of Public Health, Japan) [20]. All *p* values were two-tailed, and values of *p* < 0.05 were considered statistically significant.

## Results

### General backgrounds of laboratory-confirmed cases of COVID-19 in Vietnam as of 31 July 2020

In Vietnam, the first two cases of COVID-19 occurred among Chinese travelers and were reported on 23 January 2020. As of 31 July 2020, a total of 558 confirmed cases of COVID-19 had been reported to the WHO [15]. Background data on 544 cases were available and are shown in Table 1.

**Table 1:** Characteristics of laboratory-confirmed cases of COVID-19 in Vietnam as of 31 July 2020

Variable	
Age—median (IQR), years	35 (26–51)
Gender—male, n (%)	298 (54.8)
Nationality, n (%)	
Vietnamese	473 (86.9)
Other than Vietnam	71 (13.1)
Occupation, n (%) (n = 189)	
Medical professionals	31 (16.4)
Office workers	28 (14.8)
Driver	2 (1.1)
Student (domestic)	7 (3.7)
Student (international)	43 (22.8)
Service/commercial	14 (7.4)
Staff of airline company	5 (2.6)
School teacher	6 (3.2)
Tourist	30 (15.9)
Farmer	3 (1.6)
Specialist	12 (6.3)
Others	7 (3.7)
Determination of infection by quarantining	277 (50.9)
Epidemic cause	
Imported cases from China	7 (1.3)
Close contacted with case from China	9 (1.7)
Imported cases from other than China	326 (59.9)
Close contacted cases with cases from other than China	30 (5.5)
Infected communities	117 (21.5)
Hospital clusters	35 (6.4)
Close contacted with cases in communities	20 (3.7)
Clinical outcomes (at the time of July 31)	
Discharge from hospital	366 (67.3)
Death	3 (0.6)
In-hospital	175 (32.2)
Duration of hospitalization, median (IQR), days (n = 363)	19 (15–25)

n = 544

The median age of patients with COVID-19 was 35 years (interquartile range [IQR], 26–51 years) and 54.8% were men. Although most cases occurred among Vietnamese nationals, 13.1% of cases were of other nationalities (China, the USA, the UK, Germany, France, and Russia). The most common professions were students studying outside of Vietnam (22.8%), followed by medical professionals (16.4%) and tourists (15.9%). In over half of cases, infection was identified at the time of quarantining. The median duration of hospitalization was 19 days (IQR, 15–25 days).

### Temporal and spatial distribution of the number of COVID-19 cases and related events in Vietnam

**Fig. 1:**
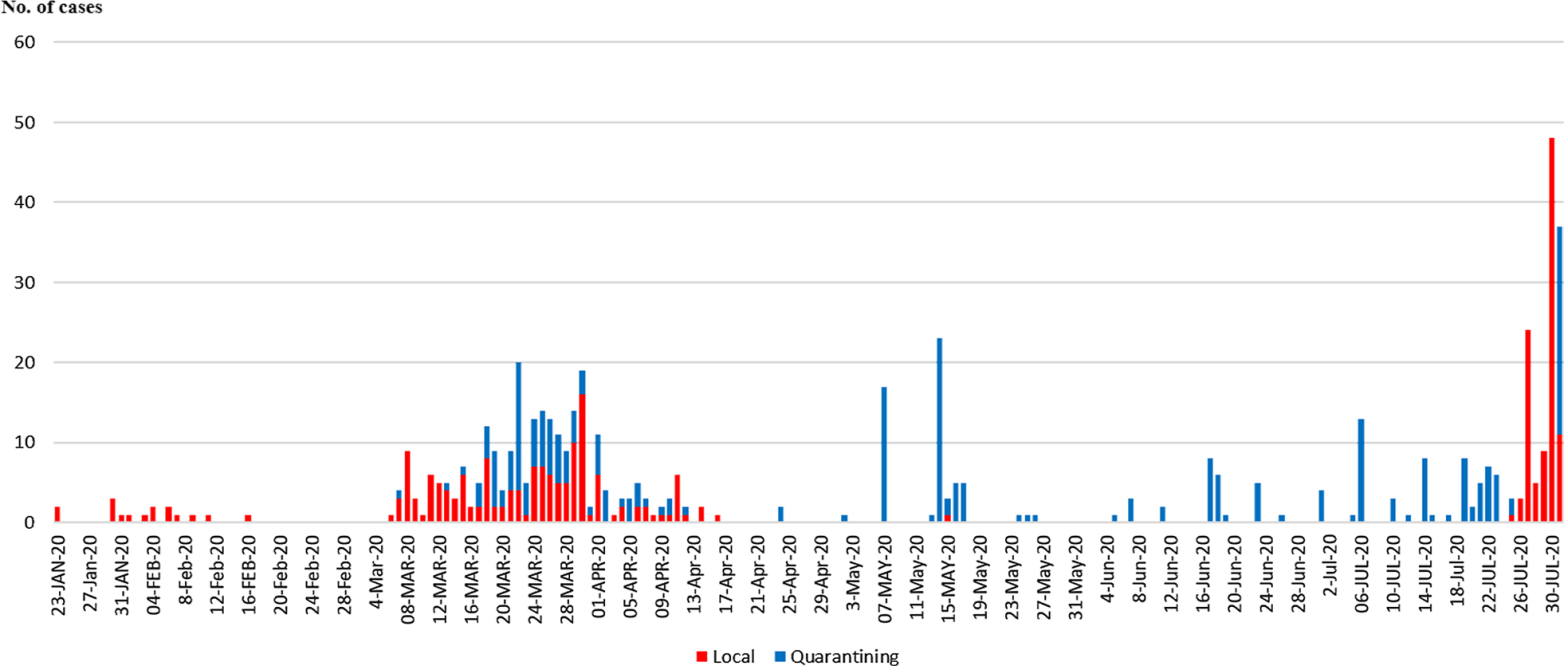
Temporal distribution of the number of daily COVID-19 cases reported as of 31 July 2020. Red bars show the numbers of cases confirmed by local authorities; blue bars show the numbers of cases confirmed during airport quarantine

Temporal changes in daily cases of COVID-19 in Vietnam are shown in Fig. 1.

After the first two cases of COVID-19 were reported in January 21, 2020 in Vietnam, the COVID-19 cases were sporadically observed until the end of February including the occurrence of a spatial cluster in Vinh Phuc Province (the first phase). Then, the epidemic wave occurred from the 1st week of March to the middle of April (the second phase). The cases in the second phases were mainly observed in Hanoi and HCMC. However, from the middle of April, only quarantined cases were reported with no local cases for 99 days had (the third phase). Finally, the second epidemic wave started on 25 July, including an outbreak (spatial cluster) in Da Nang (the fourth phase).

Information on the COVID-19 outbreak and related events in Vietnam are included as a Additional file 1: Fig. S1. The Vietnam Ministry of Health issued guidelines on COVID-19 prior to the occurrence of the first case in Vietnam. Once the first two cases of COVID-19 were reported, Vietnam cancelled all international flights to Wuhan, China. National measures were implemented following a declaration of emergency, including prohibiting flights from Vietnam to China, Taiwan, Macau, and Hong Kong. The requirement for individuals arriving in Vietnam from abroad to quarantine began on 26 February 2020.

**Fig. 2:**
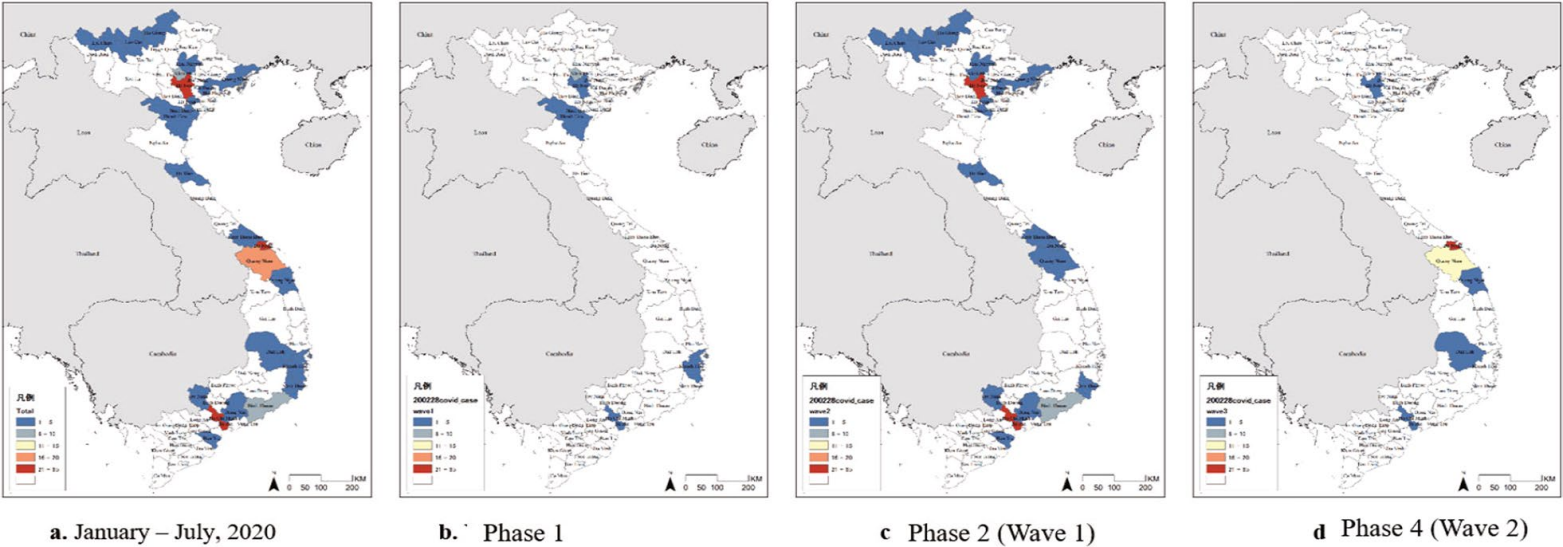
Geographical distribution of COVID-19 cases in Vietnam. **a** All phases of the outbreak, **b** Phase 1, **c** Phase 2 (Wave 1), and **d** Phase 4 (Wave 2)

The geographical distribution of COVID-19 cases in Vietnam is shown in Fig. 2.

In total, the highest number of cases was reported in Hanoi (the capital of Vietnam), followed by Da Nang (the tourist capital of South-Central Vietnam), HCMC (the most populous city and the business and financial hub of Vietnam), and Quang Nam next to Da Nang (Fig. 2a). Especially in Phase 2 (Wave 2), COVID-19 cases were reported in various provinces, including those sharing borders with mainland China (Fig. 2c). However, the number of cases in each province was small. In Wave 2, COVID-19 cases were mainly concentrated in Da Nang and Quang Nam (Fig. 2d).

### Spatial clustering of COVID-19 cases in Vietnam

**Fig. 3:**
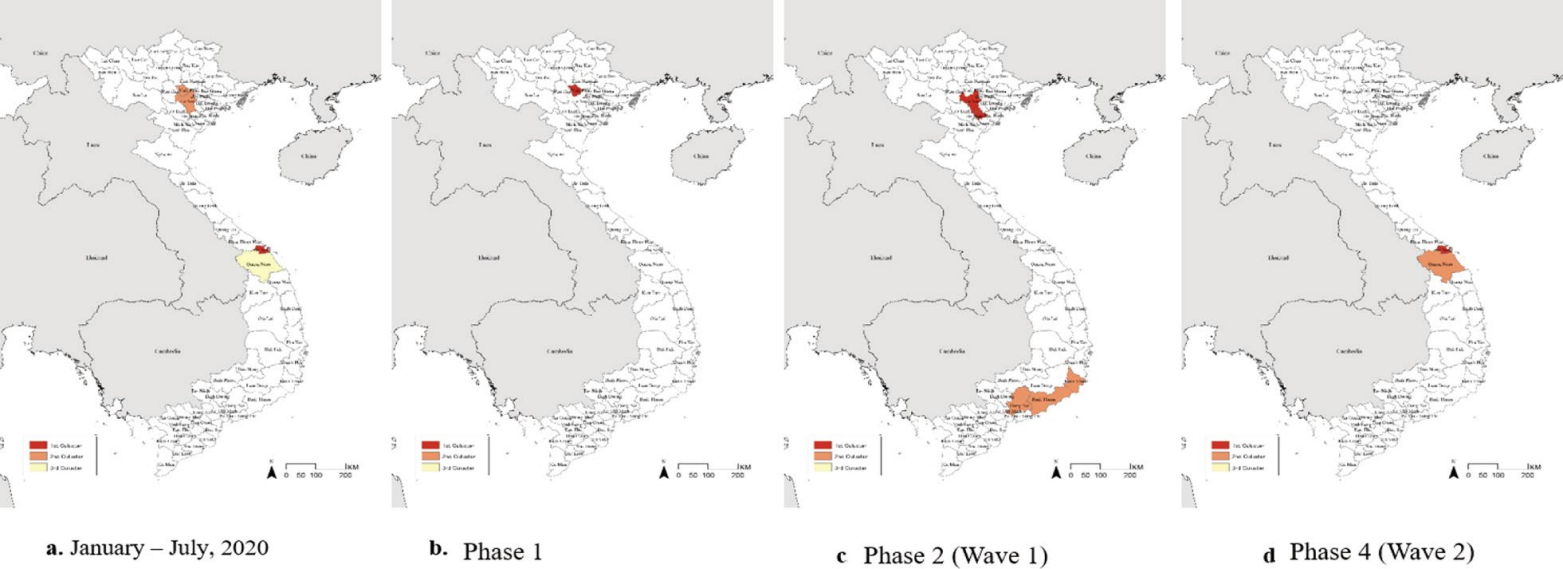
Clustering of COVID-19 cases from 23 January to 31 July 2020. **a** All phases of the outbreak, **b** Phase 1, **c** Phase 2 (Wave 1), and **d** Phase 4 (Wave 2). Red areas represent probable primary clusters of COVID-19 cases; light red areas represent secondary clusters of COVID-19 cases; and yellow areas represent tertiary clusters of COVID-19 cases

Spatial clustering of COVID-19 cases in Vietnam is shown in Fig. 3.

The most likely spatial disease cluster during the first phase of the outbreak was detected in Vinh Phuc Province (RR, 38.052; *p* < 0.001). In the second phase (Wave 1), Hanoi together with Ha Nam Province formed the most likely clusters (RR, 6.357; *p* < 0.001) (Fig. 3b), followed by Ho Chi Minh City and provinces connected to coastal areas (Dong Nai, Binh Thuan, and Ninh Thuan) as secondary clusters (RR, 1.993; *p* = 0.015) (Fig. 3c). In the fourth phase (Wave 2), the most likely spatial disease cluster was detected in Da Nang following a major outbreak (RR, 70.401; *p* < 0.001), with a secondary cluster detected in Quang Nam Province near Da Nang (RR, 7.880; *p* < 0.001) (Fig. 3d).

## Discussion

Spatial clusters of COVID-19 cases in Vietnam were mainly detected in large and populous cities as well as in cities that are hubs for business and the human mobility. In addition, the occurrence of nosocomial infections contributed to the epidemic. Quick implementation of countermeasures (including border measures and quarantine) during the early stages of the pandemic, combined with the population’s acceptance and understanding of these measures and familiarity with emerging infectious disease outbreaks, may have contributed to the overall low incidence and small numbers of severe cases of COVID-19. The age distribution of the Vietnamese population may also have contributed to the low number of severe COVID-19 cases. The results of the present study demonstrate that preparedness for future pandemics of emerging infectious diseases is crucial, especially for resource limited countries.

Vietnam was the fifth country to report COVID-19 cases outside of China after Thailand, Japan, Korea, and the USA [21]. However, Vietnam began quickly implement to countermeasures against SARS-CoV-2 at the time that China first reported an epidemic of pneumonia of unknown etiology; these included investigations, contact tracing, quarantine, and border community monitoring measures [22]. The government of Vietnam and the Vietnam Ministry of Health issued guidelines on 16 January 2020, immediately after the first imported cases from Wuhan were reported in Thailand and Japan [23, 24]. After the first two cases were identified in a hospital in HCMC, the border with China was closed, and all persons entering Vietnam were subject to a mandatory 14-day quarantine at government facilities. These quick responses were grounded in prior experience with the fight against SARS in 2003 [25, 26]. Vietnam was the country that first recognized index cases of SARS, and it was also the first to achieve successful containment among over 30 countries affected by SARS [27]. Vietnam shares a border with mainland China. Several emerging infectious disease outbreaks, including those of SARS and avian influenza H5N1, also originated in neighboring China [28]. Highly pathogenic avian influenza H5N1 virus was first isolated in Guangdong Province, China in 1996. Subsequently, human infections were reported in Hong Kong. Since 2003, outbreaks of human infection of avian influenza H5N1 have occurred in Vietnam following transmission from China [29]. As of December 2020, a total 127 cases of avian influenza H5N1 had been reported in Vietnam, the third highest number among 17 countries reporting cases, with a 50.4% case fatality rate [28]. Thus, it is possible that awareness of emerging infectious diseases originating in China was higher among the Vietnamese population compared with other countries. The present study found that during Phase 2, COVID-19 cases 2 occurred in areas bordering China in the north of Vietnam.

The first cases of COVID-19 were reported in Vietnam during the Tet holiday festival and the Lunar New Year holiday. During this period, people’s movements, distribution, trading, and consumption increase dramatically in Vietnam, China, and many other Asian countries that celebrate the Lunar New Year [30]. The quick action taken in Vietnam against COVID-19 demonstrates the importance of understanding for stopping movement of people both internationally and domestically during this period to interrupt the spread of emerging infectious diseases. It is crucial to understand social backgrounds, including local customs and behaviors, in outbreak settings to take appropriate control measures against emerging infectious diseases [14, 31, 32].

In the present study, assessment of disease clustering in Vinh Phuc Province in Phase 1 showed that the outbreak resulted from a family cluster of an individual returning from Wuhan. These cases provided novel information regarding human-to-human transmission of COVID-19 [4]. In Phase 2, a cluster occurred among students studying abroad, teachers, and flight attendants who returned to Vietnam on the same flight. In addition, spatial clustering was caused by the occurrence of nosocomial infections in a tertiary care hospital in Hanoi [33]. This hospital had experience in treating many SARS patients in 2003 without any nosocomial infections [34]. This gap may relate to the more efficient transmission of COVID-19 compared with previous coronavirus epidemics [35]. Secondary spatial clustering was detected in HCMC and neighboring areas. During this period, students studying abroad returned to Vietnam because of the COVID-19 pandemic. The students, teachers, and flight attendants who returned to Vietnam on the same flight were confirmed positive for SARS-CoV-2 during quarantine. The high transmissibility and infectivity of COVID-19, the high proportion of asymptomatic infected individuals, and the rapid and continuous spatial displacement of people all contributed to the dissemination of COVID-19 [36]. After Phase 2, no local cases occurred over the subsequent 3 months. Thereafter, some governmental measures were lifted, including re-opening of entertainment activities and domestic transportation. People who lived and worked outside of Vietnam as specialists returned to the country during this period and tested positive while in quarantine. Many of these individuals had no symptoms. This result may be compatible with those of a study showing increasing prevalence of asymptomatic patients among international travelers [37].

In Phase 3, a spatial cluster was detected only in Da Nang following a major outbreak. Da Nang is the third largest city in Vietnam, located on the coast of the South China Sea, and is a popular tourist destination. It is also a transportation hub for central Vietnam and is nearby several World Heritage Sites, including Hoi An. After the lifting of COVID-19 control measures, many people visited Da Nang and local infections were detected. Furthermore, nosocomial infections in a hospital in Da Nang played an important role in increasing the number of COVID-19 patients during the epidemic in Da Nang and was identified as the most likely spatial cluster with a high RR. A study in the US demonstrated that nosocomial COVID-19 was rare in a US large Academic Medical Center during the height of the pandemic [38], while a study conducted in January 2020 at a hospital in Wuhan suggested that 41% of patients developed the disease through nosocomial infection [39]. These results suggested that the frequency of nosocomial infections with COVID-19 may be affected by differences in the provision of medical care, available medical resources, perception of nosocomial infections by medical staff, and even hand hygiene practices [40].

The epidemic in Da Nang subsequently resulted in the first deaths in Vietnam from COVID-19. Population mobility is strongly associated with the transmission of COVID-19 and can be used as a proximal indicator to predict further outbreaks [41]. Although ethnicity has been suggested as one potential factor associated with risk of testing positive for SARS-CoV-2 and survival of COVID-19 [42], older age is also an independent risk factor for COVID-19 mortality [43]. In the present study, the median age of patients with COVID-19 in Vietnam was 35 years (IQR, 26–51 years); the observed age distribution was younger compared with previous studies, where the median age of patients was in the 40 s or 50 s [44]. In Vietnam, the average age of the national population is 31 years, and the average life expectancy is 76.3 years [45]. The younger population of Vietnam might be one factor explaining the small number of COVID-19 cases with no deaths reported during the observation period. In addition, the habit of wearing masks because of air pollution and when driving motorcycle may have been a deterrent to the spread of COVID-19. In the COVID-19 pandemic, a meta-analysis indicated that the face mask use resulted in a large reduction in risk of infection [46].

The present study had several limitations. We assessed publicly available data. Some of the data on patients’ backgrounds and clinical information were limited and were unavailable for all COVID-19 cases. The reported information and the cut-off time for the incidence of COVID-19 may have differed depending on the source of information. There may also have been a gap between the date of incidence and the real date of onset.

## Conclusions

Quick implementation of countermeasures, including quarantine and border measures, along with the people’s understanding and acceptance of these measures and close attention to emerging infectious diseases outbreaks, contributed to a relatively small number of infections and the low number of critically ill patients with COVID-19 in Vietnam. The present study indicated that attention must be paid to emerging infectious diseases prior to the emergence pandemic situations. Countermeasure implementation should be grounded in an understanding of the habits of local people and should be examined from a multidisciplinary prospective. These results warrant additional spatiotemporal analysis during the period after fatal cases of COVID-19 occurred in Vietnam.

## Supplementary Information

**Additional file 1: Fig. S1.** The COVID-19 situation and related events in Vietnam.

## Data Availability

All relevant data are included within the paper and the Additional file 1.

## Abbreviations

COVID-19: Novel coronavirus disease
2019-nCoV: 2019 Novel coronavirus
SARS-CoV-2: Severe acute respiratory syndrome coronavirus
WHO: World Health Organization
HCMC: Ho Chi Minh City
RR: Relative risk
SIR: Standardized incidence ratio
FlexScan: Flexibility shaped space–time scan statistic
IQR: Interquartile range
