# Supplementary figures and images for: Spatiotemporal distribution of COVID-19 during the first 7 months of the epidemic in Vietnam
Source: BMC Infect Dis. 2021 Oct 30;21:1124. doi: 10.1186/s12879-021-06822-0 (PMC8556820; doi:10.1186/s12879-021-06822-0)

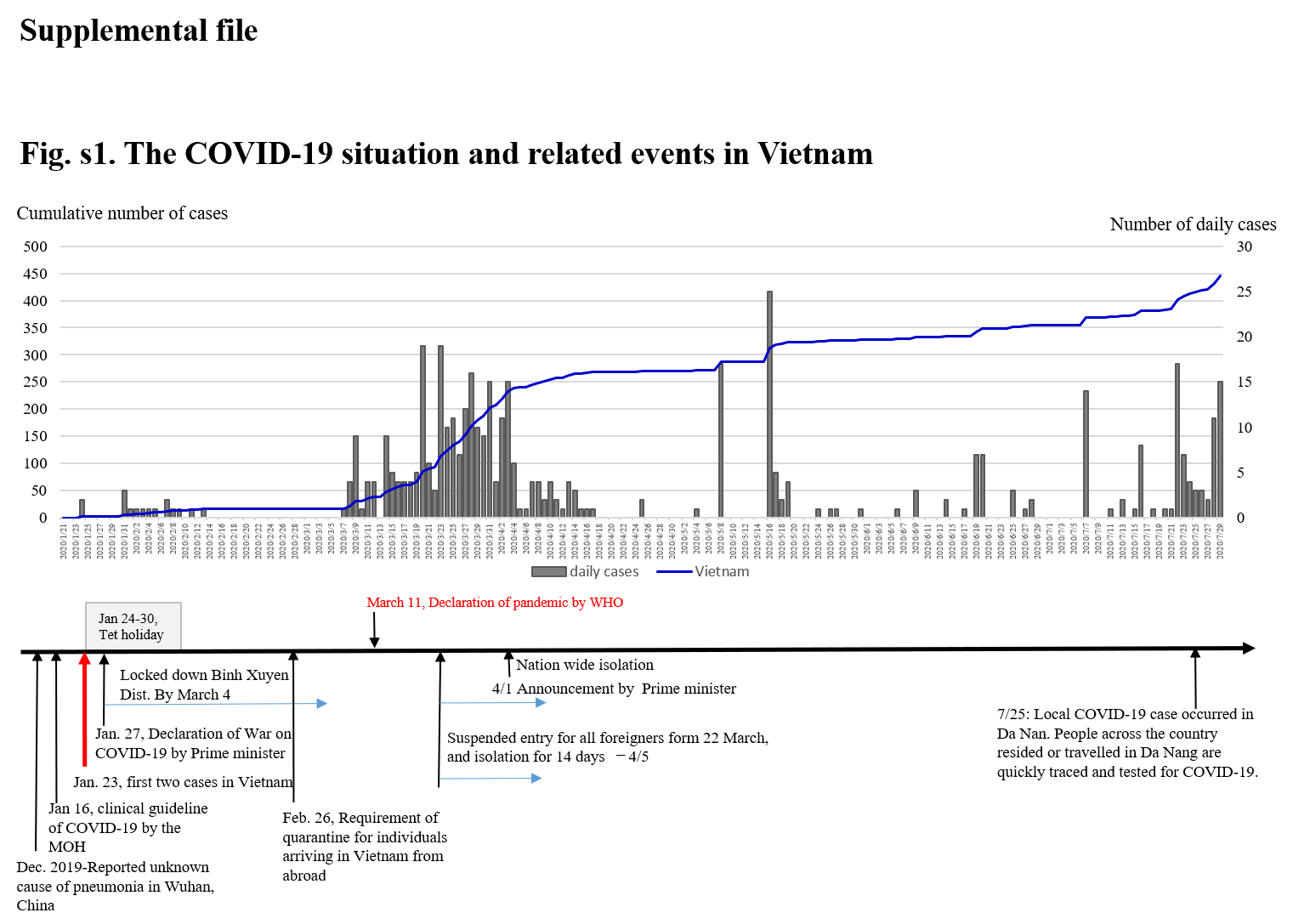

Supplement: Supplementary file 1 — Additional file 1: Fig. S1. The COVID-19 situation and related events in Vietnam. [file 12879_2021_6822_MOESM1_ESM.tif]
